# The Detection and Association of Canine Papillomavirus with Benign and Malignant Skin Lesions in Dogs

**DOI:** 10.3390/v12020170

**Published:** 2020-02-03

**Authors:** Chia-Yu Chang, Wei-Tao Chen, Takeshi Haga, Nanako Yamashita, Chi-Fen Lee, Masano Tsuzuki, Hui-Wen Chang

**Affiliations:** 1 School of Veterinary Medicine, National Taiwan University, Taipei 10617, Taiwan; flywinds11@gmail.com (C.-Y.C.); b04609022@ntu.edu.tw (W.-T.C.); chifen.lee@gmail.com (C.-F.L.); 2 Graduate Institute of Molecular and Comparative Pathobiology, School of Veterinary Medicine, National Taiwan University, Taipei 106, Taiwan; 3 Division of Infection Control and Disease Prevention, Graduate School of Agricultural and Life Sciences, The University of Tokyo, Tokyo 113-0033, Japan; ahaga@mail.ecc.u-tokyo.ac.jp (T.H.); nanakoyamashita1906@g.ecc.u-tokyo.ac.jp (N.Y.); tsuzuki-masano0615@g.ecc.u-tokyo.ac.jp (M.T.)

**Keywords:** canine papillomavirus, canine oral papillomavirus, papilloma, squamous cell carcinoma

## Abstract

Papillomavirus (PV) mainly infects the squamous epithelium and may potentially lead to benign or even malignant cutaneous lesions. However, the malignant transforming ability has been identified in several types of PVs. In humans, papillomavirus (HPV) type 16 and 18 are the most prevalent causative agents of cervical cancer. Therefore, vaccines are being developed to protect against these types. For dogs, there have been limited investigations into the association of different canine papillomavirus (CPV) genotypes with malignant lesions. Understanding the high-risk CPV genotype(s) responsible for these malignant lesions would contribute to the development of interventions for preventing CPV-induced carcinomas. In the present study, a retrospective cohort of 102 pathologically confirmed papillomas and 212 squamous cell carcinomas (SCCs) were included. The viral genome and antigens in the formalin-fixed paraffin-embedded (FFPE) tissues were detected using PCR targeting pan PV E1 and COPV L1 genes and by immunohistochemistry staining (IHC), respectively. PVs were successfully detected from 11 FFPE cutaneous tissues and four oral tissues using pan PV E1- and COPV L1-based PCR, respectively. After sequencing, CPV 1, CPV 2, and CPV 6 were detected in the benign lesions using PCR and were confirmed through IHC. While CPV 9 and CPV 15 were first detected in the SCCs of dogs, CPV 16 was most often detected in SCC specimens. The association and confirmative demonstration of viral genes and intralesional antigens of CPV 9, CPV 15, and CPV 16 in SCCs highlight the potential risk of these genotypes of CPVs in malignant transformation.

## 1. Introduction

Papillomavirus (PV) can infect and propagate in the cutaneous and mucosal epithelial cells of a wide variety of animal species with a high species specificity [1,2,3]. Although three bovine papillomaviruses (BPV 1, BPV 2, and BPV 13) have been demonstrated to cross-infect the cutaneous fibroblastic cells in equines [4,5], the majority of PVs only infect the epithelium and cause associated lesions [3,6]. To date, more than 50 genera, at least 318 types of PVs, affecting over 54 different animal species have been identified [3,7,8]. Most types of PVs cause benign proliferating skin lesions, such as warts, pigmented/viral plaques, and papillomas. However, certain types of the PVs have been confirmed as risk factors of malignant skin lesions [6]. In human medicine, human papillomavirus (HPV) types 16 and 18 are the most prevalent causative agents of the cervical cancer, as well as head and neck tumors [9]. In veterinary medicine, the bovine papillomavirus (BPV) types 1, 2, 4, and 13, and feline papillomavirus (FcaPV) types 2 and 3 have recently been demonstrated to be highly correlated to malignant neoplasms, such as squamous cell carcinoma (SCC), bowenoid in situ carcinoma (BISC) and transitional cell carcinoma [1,10,11].

Generally, the PV has a double-strained genome comprising approximately 8000 base pairs that can be generally separated into three regions—the early genes (E) encoding proteins associated with DNA replication and viral transcription; the late genes (L) controlling the expression of viral capsid proteins; and the long control region (LCR), which is associated with transcriptional factor recognition [2,6,12]. There are generally five to seven E proteins including E1, 2, 4, 5, 6, 7, and 8, which vary between types [13,14]. The L genes encode L1 and L2, the major and minor capsid proteins of PV that can assemble into the virion [3]. The PV reaches and enters the basal cells of the epithelium through microabrasions and opening wounds, then completes its life cycle and produces infective virions only when the epithelial cells undergo keratinized differentiation [2,15]. Through the differentiation procedure, the early proteins (E) are generated to manipulate the host cell cycle and achieve the viral DNA replication through different ways [15]. The over-expression of E5, E6, and E7 destroys the normal cell replication cycle, disrupts the host immune response, and influences the host gene expression, thereby contributing to the cellular transformation and oncogenicity [16,17,18].

In dogs, canine papillomaviruses (CPVs) are separated into three different genera—Lambda (types 1 and 6); Tau (types 2, 7, 13, 17, and 19); and Chi (types 3, 4, 5, 8, 9, 10, 11, 12, 14, 15, 16, and 20) genera [19,20,21]. The CPV 1, which is known as the canine oral papillomavirus (COPV), together with CPV 13, frequently forms non-neoplastic papillomas in the oral cavity of young puppies or immunosuppressed dogs [22,23]. Through contact with the infected canids, collective outbreaks of canine oral papillomatosis caused by CPV 1 have been reported in a daycare facility and a breeding farm [24,25]. Although some of the studies support that CPV 1 is unable to transform the cells, a recently published case report demonstrated that CPV 1 is highly associated with oral SCC [26,27]. Apart from CPV 1, the malignant transformations had been reported in CPV 2-, 3-, 7-, 12-, 16-, and 17-associated lesions [1,28,29]. Severe CPV 2 infection is considered to lead metastatic SCC in immunosuppressed dogs [30]. The CPV 3 has been detected in a malignant canine epidermodysplasia verruciformis [31], and the genes of CPV 7, 12, and 16 were detected in cutaneous SCCs in dogs [32]. Among these CPVs, the associations of CPV antigens or nucleic acid with malignant lesions have been demonstrated and depicted in CPV 1, CPV 2, CPV 12, and CPV 16-infected animals by using either immunohistochemical staining (IHC) or in situ hybridization [26,27,29,33].

In humans, HPV types 16 and 18 are the most prevalent causative agents of cervical cancer. Therefore, vaccines have been developed to protect against at least HPV types 16 and 18. In dogs, investigations of the association of different CPV genotypes with malignant lesions are limited. Understanding the most prevalent CPV genotype responsible for the malignant lesions would contribute to the development of interventions for preventing CPV-induced carcinomas. In the present study, 212 cases of canine SCC and 102 cases of canine papilloma (CP) collected during 2015–2018 were included. We aimed to retrospectively identify the viral DNA of CPVs by using the common general primers targeting the E1 and L1 from the formalin-fixed paraffin-embedded (FFPE) tissues that were diagnosed as SCCs or papillomas. Along with the results of PCR, the IHC was also performed by using the antigen-specific monoclonal antibody to reinforce the correlations between CPVs and the corresponding pathological lesions. This study provides further evidence of the associations between CPVs and malignant or benign cutaneous lesions in dogs.

## 2. Materials and Methods

### 2.1. Sample Collection and Ethical Approval

All clinical samples were acquired from animal hospitals in Taiwan, and the FFPE tissue blocks were kindly provided by the Graduate Institute of Molecular Comparative Pathology at National Taiwan University. The clinical samples diagnosed as canine SCC, in situ SCC, CP, or canine oral papilloma during 2015–2018 by pathologists were included and utilized in this study. All procedures involving animal sample collection were performed in accordance with the guidelines of the Institutional Animal Care and Use Committee of National Taiwan University (NTU; Taiwan, Republic of China) and were carried out under the regulations and permission of the IACUC protocol (NTU107-EL-00165) at NTU.

### 2.2. DNA Extraction from FFPE Tissue

The samples used for DNA extraction were FFPE tissues. The paraffin-embedded tissue blocks were serially sectioned to 40 μm for DNA extraction. In the de-paraffinization process, the sections were repeatedly admixed with 1.5 mL non-xylene (Muto Chemical, Tokyo, Japan) and vortexed for 15 seconds, followed by brief centrifugation at 13,000 rpm to precipitate the tissues. After the paraffin was removed, the tissue was washed twice with 1.5 mL absolute ethanol (99% (*v*/*v*), Sigma-Aldrich, St. Louis, MO, USA) and dried for 30 minutes using a heater at 37 °C, to completely remove the chemical solution. DNA was then extracted from the tissue samples using a DNeasy Blood & Tissue Kit (Qiagen, Hilden, Germany), as per the manufacturer’s instructions with some modifications. Briefly, the tissues were digested by proteinase K (Qiagen) in ATL buffer (Qiagen) using a 56 °C heater for 14–16 h. Then, the samples were transferred onto a heater at 90 °C for one hour to reverse the formaldehyde modifications of the DNA. After mixing with AL buffer (Qiagen) and absolute ethanol (99% (*v*/*v*), Sigma-Aldrich), the supernatant was applied to the specific column (Qiagen) and centrifuged until the column was empty. After washing with AW1 (Qiagen) and AW2 (Qiagen), as well as drying out the column, the DNA on the membrane of the column was eluted with 50 μL PCR-grade water. The DNA samples were stored in a refrigerator at −20 °C until use.

### 2.3. Polymerase Chain Reaction (PCR) for the Detection of CPVs

All DNA samples were amplified for CPV detection by using the general primer set—CP4/5 (forward: 5′-ATGGTACARTGGGCATWTGA-3′; reverse: 5′-GAGGYTGCAACCAAAAMTGRCT-3′) [34]. The other primer pair, COPV L1+ (5′-CTTGTTTGGGGCTTAAGAGG-3′) and COPV L1-(5′-TGCAGTGTGTACCTGTCCTG-3′) [35], was further utilized to specifically detect COPV among the SCC cases and papilloma cases which present with lesions on the oral mucosa. For each reaction, 2.5 μL of DNA, 5 μL of 10× PCR buffer (Invitrogen, Thermo Fisher Scientific, Waltham, MA, USA), 1 μL of 10 mM dNTP mixture(Invitrogen), 1.5 μL of 50 mM MgCl (Invitrogen), 1 μL of each primer (10 mM), 0.5 μL of Taq DNA polymerase (Invitrogen) and 37.5 μL of PCR-grade water were mixed. After denaturing at 94 °C for three minutes, each reaction was carried out on a PCR cycle 35 times under the following conditions—for CP4/5, 94 °C for 45 s, 55 °C for 30 s, and 72 °C for 1 min. For COPV L+/−, 94 °C for 45 s, 50 °C for 30 s, and 72 °C for 1 min. PCR ended after a 5 min final extension period. Electrophoresis was then conducted on the amplified DNA samples in 1.5% (*w*/*v*) agarose gel, which was subsequently stained with ethidium bromide (EtBr). The predicted sizes of the PCR products of each primer pair were approximately 450 bp and 280 bp for CP4/5 and COPV L+/−, respectively. The DNA quality of each sample was confirmed by the internal control PCR targeting canine GAPDH gene. The primers used in the present study were listed in Table 1.

### 2.4. Sequence Analysis

The partial gene sequences of the CPVs were sequenced and genotyped using the NCBI BLAST website (http://blast.ncbi.nlm.nih.gov/Blast.cgi). The data were analyzed and visualized using the Molecular Evolutionary Genetics Analysis Version 7.0 and Geneious software (Genewiz, Inc., Auckland, New Zealand) [36,37]. The distance measurements were conducted using DNASTAR software (Lasergene, Madison, WI, USA).

### 2.5. Immunohistochemistry Staining of Viral Antigens

The CPV-positive cases confirmed by PCR were sectioned into the 4 μm-thick slides. The slides were de-waxed in non-xylene (Muto Chemical) solution and hydrated in a graded series of ethanol from 80% to 99%. The antigen retrieval of each slide was achieved by boiling the slides at 95 °C with the Triology pretreatment solution (Cell Marque, Rocklin, CA, USA) for 10 min. Then, the slides were cooled and washed three times with PBST. Non-specific signals were blocked by incubating the slides with 10% normal goat serum for one hour at room temperature. Following washing three times with PBST buffer, mouse anti-HPV antibodies [BPV-1/1H8 + CAMVIR] (catalog no. ab2417, Abcam, Cambridge, UK) were 100-fold diluted in PBST and applied to the slides for incubation for one hour at room temperature. This antibody broadly reacts to several types of CPV, BPV, HPV, and Zalophus papillomavirus [38,39,40,41,42]. The endogenous peroxidase activity was blocked by incubating the slides with 3% H2O2 in methanol for 10 min. The goat anti-mouse antibody conjugated with horseradish peroxidase (HRP) (Dako, Agilent Technologies, Santa Clara, CA, USA) was used as the secondary antibody and incubated with the slides for one hour followed by washing three times with PBST. The coloration procedure was accomplished using DAB detection buffer (Dako, Agilent technologies). Finally, the slides were counterstained with hematoxylin for one minute, topped with a coverslip, and observed under a microscope. According to the manufacturer’s suggestion, the signals present in the nucleus, where the papillomavirus accumulates and assembles, were interpreted as positive.

## 3. Results

### 3.1. Case Information and Histological Findings

A total of 314 FFPE tissues collected from dogs were used in this study, including 212 cases of canine SCCs and 102 cases of CPs. The histopathological diagnosis of each case was confirmed by pathologists at the Graduate Institute of Molecular Comparative Pathology of the National Taiwan University. The lesion distribution of these canine SCC cases was mainly on the oral mucosa (33%), followed by head and neck skin (22%), flank (13%), limbs (12%), nasal mucosa (9%), and other locations (11%), including perianal skin, tail, lungs, thyroid glands, and lymph nodes, as well as systemic. Regarding canine papilloma, the total case number was 102, and the papilloma lesions developed mainly on the skin (76%), followed by a small portion of lesions occurring in the oral cavity (22%), and one unique case occurring in the urinary bladder. Of the 102 canine papilloma cases, intranuclear viral inclusions were observed in only 21 (21%).

### 3.2. Molecular Viral Investigation

In total, 11 positive cases for CPVs have been identified by PCR utilizing the CP4/5 primers, which target the common partial sequences of the E1 gene of CPVs. After undergoing sequence analysis and multiple sequence alignment, six types of CPVs (1, 2, 6, 9, 15, and 16) were identified (Table 2). The positive cases of CPV 1 (case no. 4), CPV 2 (case no. 5), and two CPV 6 (case no. 6 and no. 7), which were all from benign cutaneous and mucosal lesions, shared 99.2%, 98.5%, 98.9%, and 99.2% identity with the specific reference strains (GenBank accession No. NC001619.1 for CPV 1; No. AY722648.1 for CPV 2; No. FJ492744.1 for CPV 6). Two CPV 9-positive cases (case no. 8 and no. 9) collected from a viral papilloma (case no. 8) and a specimen with both SCC and papillomas (case no. 9) shared a 99.7% and 99.5% similarity with referenced CPV 9 (GenBank accession No. JF800656.1), respectively. One CPV 15-positive case (case no. 10) from a verrucous SCC shared 97.9% identity with the CPV 15 reference strain (GenBank accession No. JX899359.1). Moreover, four canine SCC cases (cases no. 11–14) also signified a CPV 16 infection. Case no. 11 (biopsied sample) and 14 (wide excised mass) chronologically came from the same patient whose lesion was diagnosed as SCC with a metastasized lymph node. The CPV 16-positive cases shared 98.7–99.5% identity with the CPV16 reference genome (GenBank accession No. KP099966.1).

On the other hand, four positive cases for CPV 1 (COPV) were identified through PCR utilizing the COPVL1+/− primers targeted on the partial sequences of the L1 gene of CPVs. The amplicon was 280 bp, and the conclusive sequences for the multiple alignments were 215 bp. Interestingly, only case no. 4 was detected by either the CP4/5 or COPVL1+/− primers. As listed in Table 2, three CPV 1 cases came from a gingival viral papilloma (case no. 1), lip viral papilloma (case no. 2), and a papilloma in the oral cavity (case no. 3). The identity of CPV1 detected in this study shared 88–100% identity with the published CPV 1 strains.

### 3.3. Phylogenetic Analysis

**Figure 1:**
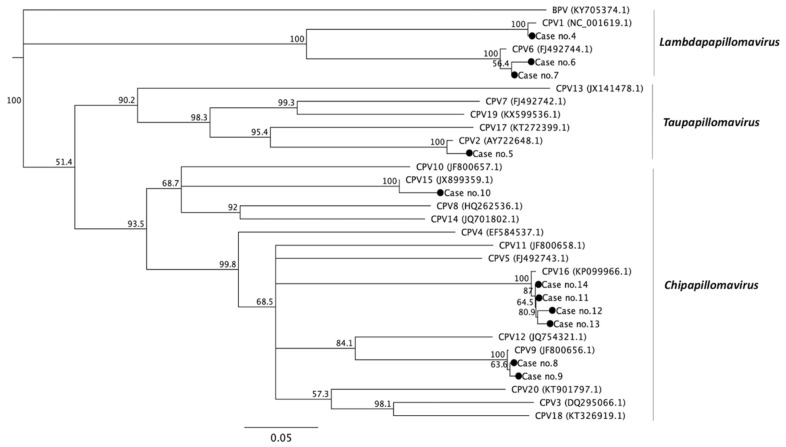
Phylogenetic analysis of the CPVs based on the partial nucleotide sequences of the E1 gene. The phylogenetic tree was analyzed by multiple sequence comparison by log-expectation (MUSCLE) and visualized using Geneious software (Genewiz, Inc.). The numbers listed at the nodes represent the percentage of 1000 bootstrap replications. The CPVs are categorized into three genera—Lambda-papillomavirus, Chi-papillomavirus, and Tau-papillomavirus. The sequences of each referenced strain of CPV were downloaded from the National Center for Biotechnology Information (NCBI) with the accession numbers listed in brackets. The CPV-positive cases identified in this study are highlighted with a solid sphere.

**Figure 2:**
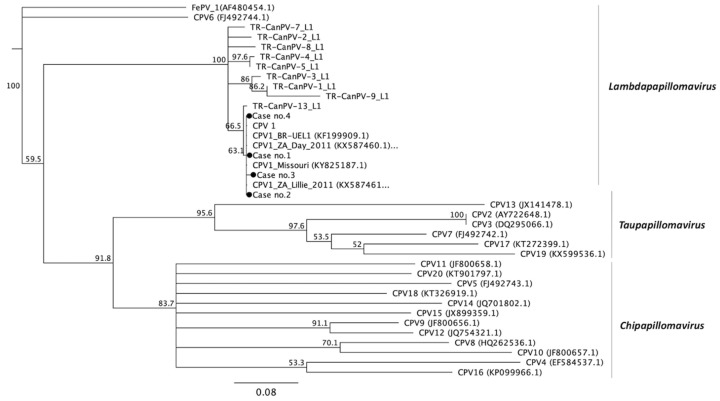
Phylogenetic analysis of CPVs based on the nucleotide sequences of the partial L1 gene. The phylogenetic tree was analyzed using multiple sequence comparison by log-expectation (MUSCLE) and visualized using Geneious software (Genewiz, Inc.). The numbers listed at the nodes represent the percentage of 1000 bootstrap replications. The CPVs are categorized into three genera—Lambda-papillomavirus, Chi-papillomavirus, and Tau-papillomavirus. The sequence of each referenced CPV strain was downloaded from the National Center for Biotechnology Information (NCBI), with the accession numbers listed in brackets. The CPV-positive cases identified in this study are highlighted with a solid sphere.

As shown in Figure 1 and Supplementary Table S1, the 20 different types of CPVs fall into three different genera [19]. The phylogenetic analysis of the partial E1 gene confirmed that case no. 5 was highly correlated with CPV 2 and belonged to the Tau-papillomavirus genera along with CPV 7, 13, 17, and 19. The sequence identity of case no. 5 with published CPV 2, 7, 13, 17, and 19 values were 98.5%, 72.1%, 62.2%, 77.9%, and 70%, respectively. The genes of case nos. 8–14 were classified into the Chi-papillomavirus genera, which included the published CPV 3, 4, 5, 8, 9, 10, 11, 12, 14, 15, 16, and 20. Case no. 8 and 9 were classified into CPV 9 with 99.7% and 99.5% similarity, respectively, and 66.5–82.7% or 66.2–82.5% similarity with the other types in the Chi-papillomavirus, respectively. Case no. 10 belonging to CPV 15 shared approximately 97.9% identity to the published CPV 15 strains and had 65.5–72.9% gene identity with the other types in the same genera. Additionally, case nos. 11–14 were classified into CPV 16 with 98.7–99.5% identity to each other, and the identities to other types in the same Chi-genera ranged from 66.0–77.4%. Case nos. 4, 6, and 7 were categorized into Lambda-papillomavirus. Case no. 4 shared approximately 99.2% and 75.3% identity with CPV 1 and CPV 6, respectively, which are the only two strains of Lambda-papillomavirus in canines. As shown in Figure 2 and Supplementary Table S2, case nos. 1, 2, 3, and 4 were categorized into Lambda-papillomavirus through PCR with COPV-specific primers (COPVL1+/−). The results demonstrate that the four cases had 87–100% sequence identity with at least 20 published L1 sequences of CPV1. The similarity of the four cases with other CPVs was quite low (47–65%) in the partial L1 gene.

### 3.4. Immunohistochemical Staining (IHC) for the Antigens of Papillomavirus

**Figure 3:**
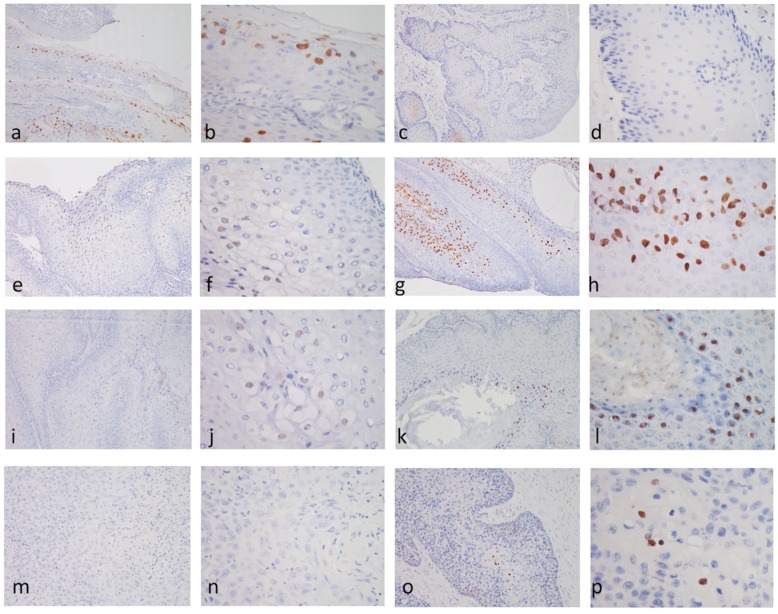
Detection of canine papillomavirus (CPV) by immunohistochemistry staining (IHC). A positive signal was identified at the intranucleus. (**a**,**b**) The representative IHC images of CPV 1 from case no. 2 with positive signals. (**c**,**d**) The representative IHC images of CPV 1 from case no. 4 with negative signals. (**e**,**f**) The representative IHC images of CPV 2 from case no. 5 with positive signals. (**g**,**h**) The representative IHC images of CPV 6 from case no. 6 with positive signals. (**I**,**j**) The representative IHC images of CPV 9 from case no. 8 with positive signals. (**k**,**l**) The representative IHC images of CPV 15 from case no. 10 with positive signals. (**m**,**n**) The representative IHC images of CPV 16 from case no. 13 with negative signals. (**o**,**p**) The IHC images of CPV 16 from case no. 11 with positive signals. The images presented for each representative case are one at low-magnitude (100×; (**a**,**c**,**e**,**g**,**I**,**k**,**m**,**o**)) and one at high-magnitude (400×; (**b**,**d**,**f**,**h**,**j**,**l**,**n**,**p**)).

The association of PV antigens with SCC and benign lesions was evaluated in all PCR-positive cases using the commercial mouse anti-HPV antibody [BPV-1/1H8 + CAMVIR]. The results for IHC are presented in Figure 3 and summarized in Table 2, where the representative images of IHC patterns of each CPV type are shown. Seven out of 14 PCR-positive cases showed strong intranuclear positive signals for IHC, including three CPV 1, one CPV 2, two CPV 6, and one CPV 15 case. Three out of 14 cases showed scattered weak positive intranuclear positive signals for IHC, including two CPV 9 and one CPV 16 case. Four out of 14 cases were completely negative for IHC, including one CPV 1 and three CPV 16 cases.

Interestingly, not all cases infected with the same type of CPV shared the same IHC patterns in this study. Among the four CPV 1-positive cases, only three were positive, while the other one was strongly negative. For CPV 16, only one case was weakly positive for IHC, and three other cases were negative.

## 4. Discussion

Clinically, most types of CPVs cause benign skin lesions, such as warts and pigmented/viral plaques or papillomas, which are self-limiting in dogs. In the present study, the viral nucleic acid has been identified, and the association of the viral antigens of several CPVs in Chi genera, including CPV 9, 15, and 16, with SCC in dogs has been demonstrated. Alongside the identified 2.3% detection rate of CPVs in SCC lesions (5/212), the results of this study highlight the risks and potential associations of CPVs in malignant transformation in dogs. Since SCC is frequently diagnosed in dogs, the oncopathogenesis of CPVs should be further investigated.

Molecular detection of the papillomavirus has been a challenge in both human and veterinary medicine due to the abundance of genotypes in the same animal species and the high heterogeneity among types [16]. At present, there is no general primer set that is able to amplify all types of PVs [43]. The degenerative primer set, CP4/5, used in the present study, is one of the most commonly used primer sets for detecting papillomavirus and searching for novel papillomaviruses. It has been successfully used to detect over 60 types of HPVs (HPVs 1–8, HPVs 10–19, HPVs 21–26, HPVs 30–38, HPV 40, HPVs 45–47, and HPV 60) [44], seven types of CPVs (CPVs 1–7) [34], four types of FcaPV (1, 3, 4, and 5), and other PVs from wild animals [42,45,46,47]. In the present study, the successful amplification of CPV 1, 2, 6, 9, 15, and CPV 16 by using the CP4/5 primers suggests the broader application of this primer set in CPVs. However, the feasibility of using CP4/5 primers in detecting the other types of CPVs (8, 10, 11, 12, 13, 14, 17, and 18) is undetermined. Therefore, infection by these types of CPVs cannot be completely ruled out in this study. Furthermore, the sample quality from FFPE tissues is also a limitation in the retrospective survey. It has been reported that only 62.7% to 73.3% of the HPV-positive cases remained positive in qPCR after the FFPE procedure [48]. Considering the two affecting factors listed above, the total percentage of CPV-positive canine papilloma and SCC cases may be underestimated.

The PVs have been claimed to be the causative agent of several types of malignant neoplasms in both human and veterinary medicine [3,6,12]. As for the canine PVs, CPV 1, 2, 3, 7, 12, 16, and 17 have been speculated to cause malignant transformation of the epithelium [1,28]. The association of viral antigens or genome with malignant lesions has also been demonstrated in CPV 1-, 2-, 12-, and 16-infected animals [26,27,29,33]. In the present study, we examined canine papillomas and canine SCCs taken from the oral cavity including the gingiva, lip, and tongue and demonstrated CPV 1 in four benign lesions. CPV 1 usually affects young dogs and causes papillomatosis and benign mucosal lesions in the oral cavity with occasional outbreaks in groups [24,25]. Several retrospective studies based on molecular or serological survey evidence have declared that CPV 1 is unlikely to bring about malignant changes [49,50,51]. Controversially, accumulative studies have revealed the identification of CPV 1 in SCCs [26,27]. Furthermore, CPV 2 was also only identified in a benign viral papilloma case in the present study. Usually, CPV 2 was detected from benign lesions, such as endophytic papillomas, exophytic papillomas, and, occasionally, in SCCs from immunosuppressed dogs [30,33]. These different findings might be due to differences among these CPV genomes, such as mutations in E5, E6, and E7 [16,17,18], or host immune responses. Further investigations should be performed to understand the oncogenesis of the CPVs.

According to the literature, CPV 9 and CPV 15 have only been reported in benign lesions [20,52]. In the present study, CPV 9 was identified in a dog with multiple cutaneous neoplasms, including multiple skin papillomas, cutaneous horn, and SCCs (case no. 9) and CPV 15 was confirmed to be associated with verrucous SCC. Our data provide the first evidence and report of CPV 9-associated SCC and CPV 15-associated premalignant cutaneous tumors in dogs. Furthermore, we also demonstrated that the viral antigen and nucleic acid of CPV 16 were identified from three SCCs and one in situ SCC. This finding echoes the speculation proposed by Luff et al., suggesting that CPV 16 is a high-risk type of CPV that brings about not only pigmented plaques but also malignant carcinomas [53].

In conclusion, the association of CPVs with benign and malignant lesions was not only demonstrated through PCR, but also by the detection of the intralesional viral antigens using IHC. To our knowledge, this is the first report providing the evidence of the association of CPV 9 and CPV 15 in malignant lesions, such as SCCs. The association and the confirmative demonstration of viral genes and intralesional antigens of CPV 9, CPV 15, and CPV 16 in SCCs highlight the potential risk of theses genotypes of CPVs in malignant transformations.

## Figures and Tables

**Table 1:** The primers used in this study.

Primer	Sequence (5′-3′)
CP4/5	Forward: 5′-ATGGTACARTGGGCATWTGA-3′ Reverse: 5′-GAGGYTGCAACCAAAAMTGRCT-3′
COPV L1+/−	Forward: 5′-CTTGTTTGGGGCTTAAGAGG-3′ Reverse: 5′-TGCAGTGTGTACCTGTCCTG-3′
GAPDH	Forward: 5′-AGGCTGAGAACGGGAAACTT-3′ Reverse: 5′-TTTGGCTAGAGGAGCCAAGC-3′

**Table 2:** Summary of the sample information and results of PCR and IHC. The case number, breed, and patient age of the canine papillomavirus (CPV)-positive cases are listed. Information regarding the lesions and histopathological diagnosis of the case, the corresponding types of CPVs detected by PCR, the gene identity, and immunohistochemical staining (IHC) results are also summarized. The accession number of each sequence from GenBank is also provided. NE, the clinician did not provide information for this case; y, year-old; +, clear, intranuclear positive signal shown in IHC; −, completely negative in IHC.

Case No.	Breed	Age	Location	Histopathological Diagnosis	Type of PVs	Gene Identity	IHC	Accession No.
1	Mixed	5y	Gingiva	Viral papilloma	CPV 1	100%	+	MN617831
2	Mixed	5y	Lip	Viral papilloma	CPV 1	100%	+	MN617832
3	Corgi	NE	Oral	Papilloma	CPV 1	99.5%	+	MN617833
4	Poodle	6y	Digital Lip	Papilloma Acanthosis	CPV 1	99.5%	-	MN617834 MN586852
5	Golden retriever	8y	Elbow	Viral papilloma	CPV 2	98.5%	+	MN606026
6	Pomeranian	6y	Digital	Inverted papilloma	CPV 6	97.4%	+	MN606027
7	Schnauzer	7y	Paw	Viral papilloma	CPV 6	99.2%	+	MN606028
8	Unknown	9y	Digital	Viral papilloma	CPV 9	99.7%	+	MN606029
9	Schnauzer	11y	Inguinal Flank	Squamous cell carcinoma (SCC) Cutaneous horn Papilloma	CPV 9	99.5%	+	MN606030
10	Poodle	7y	Digital	Verrucous squamous cell carcinoma	CPV 15	97.9%	+	MN606031
11	Mixed	6y	Inguinal	Squamous cell carcinoma (SCC)	CPV 16	99.5%	+	MN606032
12	Maltese	8y	Gingiva	Dysplasia of squamous epithelium	CPV 16	98.7%	-	MN606033
13	Golden retriever	14y	Sublingual	Squamous cell carcinoma (SCC)	CPV 16	98.7%	-	MN606034
14	Mixed	6y	Inguinal	Squamous cell carcinoma (SCC)	CPV 16	99.5%	-	MN606035

## Supplementary Materials

The following are available online at https://www.mdpi.com/1999-4915/12/2/170/s1: Supplementary Table S1: The pairwise sequence identity matrix of partial E1 genes of canine papillomaviruses (CPVs); Supplementary Table S2: The pairwise sequence identity matrix of partial L1 genes of canine papillomaviruses (CPVs).
