# Supplementary material for: The Detection and Association of Canine Papillomavirus with Benign and Malignant Skin Lesions in Dogs
Source: Viruses. 2020 Feb 3;12(2):170. doi: 10.3390/v12020170 (PMC7077320; doi:10.3390/v12020170)
Supplement: Supplementary file 1 [file viruses-12-00170-s001.pdf]

Supplementary Table

Table S1. The pairwise sequence identity matrix of partial E1 genes of canine papillomaviruses (CPVs). The identity of partial E1 gene of CPVs were analyzed by directly comparing the sequences without considering the phylogenetic relationship and present in percentage (%). The reprehensive CPVs with their accession numbers on GenBank of each type were provided.

Percent Identity (%)																																
		1	2	3	4	5	6	7	8	9	10	11	12	13	14	15	16	17	18	19	20	21	22	23	24	25	26	27	28	29	30	31
BPV (KY705374.1)	1																															
CPV1 (NC001619.1)	2	55.4																														
CPV2 (AY722648.1)	3	54.5	60.3																													
CPV3 (DQ295066.1)	4	54.2	53.8	62.1																												
CPV4 (EF584537.1)	5	54.2	61.4	61.0	72.1																											
CPV5 (FJ492743.1)	6	55.8	58.0	59.2	78.2	76.3																										
CPV6 (FJ492744.1)	7	54.5	75.7	61.6	56.3	61.8	58.2																									
CPV7 (FJ492742.1)	8	55.5	55.6	72.7	62.8	60.2	59.4	59.9																								
CPV8 (HQ262536.1)	9	58.4	60.3	60.0	66.3	71.1	67.1	57.6	59.4																							
CPV9 (JF800656.1)	10	56.6	54.8	59.7	75.3	73.2	76.1	59.2	58.6	65.5																						
CPV10 (JF800657.1)	11	58.7	60.6	61.0	69.5	70.8	69.5	59.5	61.5	74.7	65.5																					
CPV11 (JF800658.1)	12	56.6	54.8	61.6	75.3	74.5	76.6	58.9	60.2	67.4	74.7	68.2																				
CPV12 (JQ754321.1)	13	55.3	56.9	61.0	76.3	75.8	79.7	60.0	58.4	69.2	82.4	67.1	73.9																			
CPV13 (JX141478.1)	14	53.9	53.1	62.9	56.8	57.8	54.4	53.1	60.5	57.3	56.5	59.1	57.8	55.5																		
CPV14 (JQ701802.1)	15	56.1	62.4	60.8	66.1	72.1	67.1	62.4	58.6	78.4	67.4	72.4	69.2	68.4	57.1																	
CPV15 (JX899359.1)	16	60.5	60.6	64.2	68.9	68.9	67.6	61.8	63.9	74.5	67.4	74.5	69.5	68.4	57.4	73.8																
CPV16 (KP099966.1)	17	57.9	59.5	57.9	71.3	71.8	75.8	60.5	58.6	66.6	76.8	66.3	75.8	76.1	57.7	66.1	68.5															
CPV17(KT272399.1)	18	55.1	57.9	77.3	60.0	61.0	58.7	59.5	70.6	59.7	60.3	62.1	61.0	58.4	62.9	59.8	60.4	57.5														
CPV18 (KT326919.1)	19	54.2	56.7	60.3	83.7	73.7	77.4	57.4	57.6	65.8	75.0	69.5	75.8	75.0	55.1	66.9	69.3	69.6	58.0													

CPV19 (KX599536.1)	**20**	57.7	59.0	70.8	59.0	62.9	59.2	57.7	78.4	59.7	59.7	60.0	60.8	59.0	62.0	60.5	61.0	58.4	72.8	58.4												
CPV20 (KX599536.1)	**21**	53.4	53.5	58.2	77.1	77.1	78.4	54.5	58.9	66.6	77.1	67.4	76.8	75.5	55.1	68.0	68.5	71.9	59.3	79.0	58.4											
Case no. 4	**22**	55.6	99.2	60.3	54.1	61.2	58.3	75.3	55.9	60.4	54.6	61.2	54.3	57.0	53.5	63.1	61.0	59.2	58.1	57.1	58.7	53.7										
Case no. 5	**23**	54.3	59.7	98.4	61.6	60.5	58.7	61.3	71.6	59.5	59.2	60.5	61.3	60.5	62.2	60.4	63.7	57.5	77.1	59.8	69.8	57.8	59.9									
Case no. 6	**24**	54.6	74.7	60.8	55.3	60.8	57.1	98.9	59.2	57.1	58.2	58.7	57.9	58.9	53.4	61.7	60.9	59.6	58.8	56.4	56.9	53.5	74.8	60.6								
Case no. 7	**25**	54.9	74.9	61.0	56.1	61.3	57.6	99.2	59.7	57.6	58.9	59.5	58.4	59.7	54.2	62.2	61.7	60.4	59.1	57.0	57.4	54.1	75.1	60.9	99.2							
Case no. 8	**26**	56.3	54.8	59.7	75.5	73.2	76.1	58.9	58.4	65.8	99.7	65.3	74.5	82.4	56.6	67.2	67.2	76.6	60.4	75.3	59.7	76.9	54.7	59.3	57.7	58.8						
Case no. 9	**27**	56.1	54.6	59.5	75.3	72.9	75.8	58.7	58.1	65.5	99.5	65.0	74.2	82.1	56.4	66.9	66.9	76.4	60.1	75.1	59.5	76.6	54.5	59.1	57.7	58.5	99.7					
Case no. 10	**28**	59.4	59.0	63.1	67.1	67.4	65.5	60.0	62.6	72.9	65.8	72.6	67.6	66.8	56.5	72.2	97.9	66.4	58.8	67.5	59.7	66.9	59.7	62.7	60.6	60.4	65.6	65.6				
Case no. 11	**29**	58.2	59.5	58.2	70.8	72.1	75.8	60.5	59.2	66.6	76.8	66.3	75.3	76.1	58.2	65.6	68.8	99.5	57.5	70.1	58.7	71.9	59.2	57.8	59.5	60.4	76.6	76.4	66.7			
Case no. 12	**30**	57.9	58.7	57.7	70.0	71.3	75.0	59.7	58.6	66.1	76.1	65.8	74.5	75.3	57.7	65.4	68.0	98.7	57.0	69.3	58.2	71.1	58.6	57.3	59.8	60.4	75.9	75.9	66.7	99.2		
Case no. 13	**31**	58.4	58.7	57.4	70.3	71.3	75.3	59.7	58.6	66.6	76.1	66.1	74.7	75.3	57.7	65.4	68.2	98.7	56.7	69.6	58.2	71.4	58.9	57.0	59.8	60.1	75.9	75.6	66.7	99.2	98.7	
Case no.14	**32**	58.2	59.5	58.2	70.8	72.1	75.8	60.5	59.2	66.6	76.8	66.3	75.3	76.1	58.2	65.6	68.8	99.5	57.5	70.1	58.7	71.9	59.2	57.8	59.5	60.4	76.6	76.4	66.7	100	99.2	99.2

Table S2. The pairwise sequence identity matrix of partial L1 genes of canine papillomaviruses (CPVs). The identity of partial L1 gene of CPVs were analyzed by directly comparing the sequences without considering the phylogenetic relationship and present in percentage (%). The reprehensive CPVs with their accession numbers on GenBank of each type were provided.

**Percent Identity (%)**																							
		**1**	**2**	**3**	**4**	**5**	**6**	**7**	**8**	**9**	**10**	**11**	**12**	**13**	**14**	**15**	**16**	**17**	**18**	**19**	**20**	**21**	**22**
**FePV_1(AF480454.1)**	**1**																						
**CPV 1(NC001619.1)**	**2**	64.2																					
**CPV1_BR-UEL1 (KF199909.1)**	**3**	64.2	100.0																				
**CPV1_Missouri (KY825187.1)**	**4**	64.2	100.0	100.0																			

**CPV1_ZA_Day_2011 (KX587460.1)**	**5**	64.2	100.0	100.0	100.0																		
**CPV1_ZA_Lillie_2011 (KX587461.1)**	**6**	64.2	100.0	100.0	100.0	100.0																	
**TR-CanPV-1_L1**	**7**	63.7	93.9	93.9	93.9	93.9	93.9																
**TR-CanPV-2_L1**	**8**	64.7	97.6	97.6	97.6	97.6	97.6	94.3															
**TR-CanPV-3_L1**	**9**	62.8	94.8	94.8	94.8	94.8	94.8	95.3	95.3														
**TR-CanPV-4_L1**	**10**	64.7	96.7	96.7	96.7	96.7	96.7	93.9	96.2	93.4													
**TR-CanPV-5_L1**	**11**	64.7	97.2	97.2	97.2	97.2	97.2	93.9	96.7	93.9	99.5												
**TR-CanPV-7_L1**	**12**	64.7	98.1	98.1	98.1	98.1	98.1	94.8	98.6	96.7	96.7	97.2											
**TR-CanPV-8_L1**	**13**	64.2	97.2	97.2	97.2	97.2	97.2	93.4	97.2	95.3	95.3	95.8	97.6										
**TR-CanPV-9_L1**	**14**	59.1	87.7	87.7	87.7	87.7	87.7	93.9	88.2	92.0	87.7	87.7	89.6	88.2									
**TR-CanPV-13_L1**	**15**	64.2	99.1	99.1	99.1	99.1	99.1	93.9	97.6	95.8	96.7	97.2	98.1	97.2	88.7								
**CPV2 (AY722648.1)**	**16**	49.8	50.0	50.0	50.0	50.0	50.0	50.5	48.6	50.0	50.0	50.0	50.0	48.6	49.1	50.0							
**CPV3 (DQ295066.1)**	**17**	49.8	50.0	50.0	50.0	50.0	50.0	50.5	48.6	50.0	50.0	50.0	50.0	48.6	49.1	50.0	100.0						
**CPV4 (EF584537.1)**	**18**	51.0	49.3	49.3	49.3	49.3	49.3	47.3	48.8	48.3	47.3	47.8	48.3	48.3	46.4	48.8	44.9	44.9					
**CPV5 (FJ492743.1)**	**19**	51.0	51.2	51.2	51.2	51.2	51.2	51.2	51.2	50.2	50.7	51.2	50.2	50.2	48.8	51.2	51.2	51.2	57.1				
**CPV6 (FJ492744.1)**	**20**	69.3	65.6	65.6	65.6	65.6	65.6	63.7	63.7	63.3	64.2	64.7	64.7	64.2	59.5	65.1	54.4	54.4	49.0	54.8			
**CPV7 (FJ492742.1)**	**21**	49.3	52.8	52.8	52.8	52.8	52.8	51.4	51.4	51.9	51.9	52.4	52.8	51.4	49.1	52.8	70.9	70.9	48.3	53.6	51.6		
**CPV8 (HQ262536.1)**	**22**	53.0	51.4	51.4	51.4	51.4	51.4	50.5	50.5	50.9	50.5	50.9	50.5	50.5	47.2	50.5	49.3	49.3	59.2	63.5	53.5	48.8	
**CPV9 (JF800656.1)**	**23**	52.8	54.5	54.5	54.5	54.5	54.5	55.0	55.0	54.5	55.0	55.5	54.5	53.6	52.6	54.5	48.8	48.8	52.4	64.1	54.7	49.8	62.5
**CPV10 (JF800657.1)**	**24**	51.6	48.1	48.1	48.1	48.1	48.1	47.2	48.1	47.6	47.2	47.6	48.1	47.2	45.8	47.2	48.3	48.3	60.2	60.6	48.4	50.2	70.7
**CPV11 (JF800658.1)**	**25**	52.6	53.9	53.9	53.9	53.9	53.9	53.4	52.9	53.9	52.4	52.9	53.9	53.9	52.4	53.9	54.9	54.9	57.1	64.9	50.7	55.9	56.6
**CPV12 (JQ754321.1)**	**26**	53.8	53.6	53.6	53.6	53.6	53.6	54.5	54.1	54.5	53.1	53.6	53.6	53.1	51.2	53.6	48.3	48.3	54.9	62.1	51.4	49.3	62.0
**CPV13 (JX141478.1)**	**27**	52.1	50.0	50.0	50.0	50.0	50.0	51.9	50.5	50.9	50.9	51.4	50.9	49.1	49.1	49.5	54.4	54.4	46.6	50.7	49.8	59.2	46.7

**CPV14 (JQ701802.1)**	**28**	49.3	51.9	51.9	51.9	51.9	51.9	52.8	51.9	51.9	51.4	51.4	52.4	51.9	50.9	51.9	51.2	51.2	54.7	59.6	50.2	51.2	67.3
**CPV15 (JX899359.1)**	**29**	57.7	52.8	52.8	52.8	52.8	52.8	53.3	53.8	52.4	52.4	52.8	53.3	52.4	50.9	52.8	50.5	50.5	53.9	56.8	56.7	51.0	61.5
**CPV16 (KP099966.1)**	**30**	54.3	51.7	51.7	51.7	51.7	51.7	51.2	51.2	50.2	50.7	50.7	50.7	50.7	50.7	51.7	51.2	51.2	63.5	67.5	51.9	52.2	61.1
**CPV17 (KT272399.1)**	**31**	47.9	49.5	49.5	49.5	49.5	49.5	49.5	47.2	49.5	48.1	48.1	48.6	47.2	49.1	48.6	71.9	71.9	49.8	50.2	54.4	73.9	52.6
**CPV18 (KT326919.1)**	**32**	56.6	56.0	56.0	56.0	56.0	56.0	56.9	55.5	56.5	55.5	56.0	55.0	55.5	54.5	56.0	48.8	48.8	57.0	67.6	57.5	45.0	60.8
**CPV19 (KX599536.1)**	**33**	46.5	48.1	48.1	48.1	48.1	48.1	46.7	45.8	46.7	46.7	47.2	46.7	45.8	44.3	47.2	65.7	65.7	46.8	50.2	50.2	72.5	49.8
**CPV20 (KT901797.1)**	**34**	54.5	54.4	54.4	54.4	54.4	54.4	55.8	53.4	54.9	53.9	53.9	53.9	53.4	53.9	54.4	52.4	52.4	57.3	65.0	54.1	51.9	62.1
**Case no.1**	**35**	64.2	100.0	100.0	100.0	100.0	100.0	93.9	97.6	94.8	96.7	97.2	98.1	97.2	87.7	99.1	50.0	50.0	49.3	51.2	65.6	52.8	51.4
**Case no.2**	**36**	64.2	100.0	100.0	100.0	100.0	100.0	93.9	97.6	94.8	96.7	97.2	98.1	97.2	87.7	99.1	50.0	50.0	49.3	51.2	65.6	52.8	51.4
**Case no.3**	**37**	63.2	99.5	99.5	99.5	99.5	99.5	93.4	97.2	94.3	96.2	96.7	97.6	96.7	87.3	98.6	50.0	50.0	49.3	51.2	65.1	52.8	50.9
**Case no.4**	**38**	64.1	99.5	99.5	99.5	99.5	99.5	93.4	97.2	94.3	96.2	96.7	97.6	96.7	87.3	98.6	49.5	49.5	49.3	51.2	65.6	52.4	51.4

**Percent Identity (%)**																	
		**23**	**24**	**25**	**26**	**27**	**28**	**29**	**30**	**31**	**32**	**33**	**34**	**35**	**36**	**37**	**38**
**FePV_1(AF480454.1)**	**1**																
**CPV 1(NC001619.1)**	**2**																
**CPV1_BR-UEL1 (KF199909.1)**	**3**																
**CPV1_Missouri (KY825187.1)**	**4**																
**CPV1_ZA_Day_2011 (KX587460.1)**	**5**																
**CPV1_ZA_Lillie_2011 (KX587461.1)**	**6**																
**TR-CanPV-1_L1**	**7**																

**TR-CanPV-2_L1**	**8**																
**TR-CanPV-3_L1**	**9**																
**TR-CanPV-4_L1**	**10**																
**TR-CanPV-5_L1**	**11**																
**TR-CanPV-7_L1**	**12**																
**TR-CanPV-8_L1**	**13**																
**TR-CanPV-9_L1**	**14**																
**TR-CanPV-13_L1**	**15**																
**CPV2 (AY722648.1)**	**16**																
**CPV3 (DQ295066.1)**	**17**																
**CPV4 (EF584537.1)**	**18**																
**CPV5 (FJ492743.1)**	**19**																
**CPV6 (FJ492744.1)**	**20**																
**CPV7 (FJ492742.1)**	**21**																
**CPV8 (HQ262536.1)**	**22**																
**CPV9 (JF800656.1)**	**23**																
**CPV10 (JF800657.1)**	**24**	63.0															
**CPV11 (JF800658.1)**	**25**	67.0	59.0														
**CPV12 (JQ754321.1)**	**26**	83.0	64.9	71.4													
**CPV13 (JX141478.1)**	**27**	51.2	49.5	51.5	50.2												
**CPV14 (JQ701802.1)**	**28**	56.7	63.4	60.5	58.2	46.2											
**CPV15 (JX899359.1)**	**29**	57.7	63.0	55.4	57.2	51.7	58.7										
**CPV16 (KP099966.1)**	**30**	60.7	57.6	59.0	64.6	45.4	60.1	59.7									
**CPV17 (KT272399.1)**	**31**	52.6	51.2	49.0	50.7	55.8	50.2	49.5	49.3								
**CPV18 (KT326919.1)**	**32**	72.2	58.9	64.6	67.5	49.8	56.5	58.9	63.3	48.8							

**CPV19 (KX599536.1)**	**33**	52.6	47.4	53.9	49.8	57.3	50.2	50.5	48.8	71.1	48.3						
**CPV20 (KT901797.1)**	**34**	65.5	60.7	64.6	68.0	48.1	58.7	58.7	62.6	54.9	67.0	46.1					
**Case no.1**	**35**	54.5	48.1	53.9	53.6	50.0	51.9	52.8	51.7	49.5	56.0	48.1	54.4				
**Case no.2**	**36**	54.5	48.1	53.9	53.6	50.0	51.9	52.8	51.7	49.5	56.0	48.1	54.4	100.0			
**Case no.3**	**37**	54.1	47.6	53.9	54.1	50.5	52.4	53.3	51.7	50.0	55.5	48.6	54.9	99.5	99.5		
**Case no.4**	**38**	54.5	48.1	53.4	53.1	50.0	51.9	52.8	51.7	49.5	56.0	48.1	54.4	99.5	99.5	97.0	
